# Heart and Lungs Protection Technique for Cardiac Surgery with Cardiopulmonary Bypass

**DOI:** 10.5195/cajgh.2014.169

**Published:** 2014-12-12

**Authors:** Vladimir Pichugin, Nikolay Melnikov, Farkhad Olzhayev, Alexander Medvedev, Sergey Jourko, Alishir Gamzaev, Vladimir Chiginev

**Affiliations:** 1 Nizhny Novgorod State Medical Academy, Russia; 2 Cardiac and Vascular Surgery Centre, Nizhny Novgorod, Russia; 3 Center for Life Sciences, Nazarbayev University, Astana, Kazakhstan

**Keywords:** constant coronary perfusion, pulmonary artery perfusion, cardiopulmonary bypass

## Abstract

**Introduction:**

Cardioplegic cardiac arrest with subsequent ischemic-reperfusion injuries can lead to the development of inflammation of the myocardium, leucocyte activation, and release of cardiac enzymes. Flow reduction to the bronchial arteries, causing low-flow lung ischemia, leads to the development of a pulmonary regional inflammatory response. Hypoventilation during cardiopulmonary bypass (CPB) is responsible for development of microatelectasis, hydrostatic pulmonary edema, poor compliance, and a higher incidence of infection. Based on these facts, prevention methods of these complications were developed. The aim of this study was to evaluate constant coronary perfusion (CCP) and the “beating heart” in combination with pulmonary artery perfusion (PAP) and “ventilated lungs” technique for heart and lung protection in cardiac surgery with CPB.

**Methods:**

After ethical approval and written informed consent, 80 patients undergoing cardiac surgery with normothermic CPB were randomized in three groups. In the first group (22 patients), the crystalloid cardioplegia without lung ventilation/perfusion techniques were used. In the second group (30 patients), the CCP and “beating heart” without lung ventilation/perfusion techniques were used. In the third group (28 patients), the CCP with PAP and lung ventilation techniques were used. Clinical, functional parameters, myocardial damage markers (CK MB level), oxygenation index, and lung compliance were investigated.

**Results:**

There were higher rates of spontaneous cardiac recovery and lower doses of inotrops in the second and third groups. Myocardial contractility function was better preserved in the second and third groups. The post-operative levels of CK-MB were lower than in control group. Three hours after surgery CK-MB levels in the second and third groups were lower by 38.1% and 33.3%, respectively. Eight hours after surgery, CK-MB levels were lower in the second and third groups by 45.9% and 47.7%, respectively. 24 hours after surgery, CK-MB levels were lower in the second and third groups by 42.0% and 42.6%, respectively, and lower by 29.7% and 27.4% 48 hours after surgery, respectively. Normalization of CK-MB levels were registered earlier in second and third groups (within 24 hours) than the control group. Oxygenation index and lung compliance were significantly higher in the third group after CPB.

**Conclusion:**

Our technique improved myocardial and lung function in patients, but larger prospective randomized trials are needed to definitively assess the protective effects of this technique.

